# The complete chloroplast genome of alien invasive species, *Diodia virginiana* L. (rubiaceae), in China

**DOI:** 10.1080/23802359.2019.1670110

**Published:** 2019-09-24

**Authors:** Han Xu

**Affiliations:** Institute of Plant Quarantine, Chinese Academy of Inspection and Quarantine, Beijing, China

**Keywords:** Chloroplast genome, *Diodia virginiana*, *Spermacoceae*, *Rubiaceae*, China

## Abstract

The first complete chloroplast genome sequences of new intercepted alien weeds in China, *Diodia*, were reported in this study. The *D. virginiana* plastome was 154,387 bp long, with the large single copy (LSC) region of 83,625 bp, the small single copy (SSC) region of 17,475 bp, and two inverted repeat (IR) regions of 26,643 bp. The plastome contained 137 genes, including 92 proteincoding, eight ribosomal RNA, and 37 transfer RNA genes. The overall GC content was 37.2%. Phylogenetic analysis of 24 representative plastomes within the order Gentianales suggests that subfamily Cinchonoideae is closely related to subfamily Ixoroideae compared with subfamily Rubioideae in Rubiaceae. Tribe Spermacoceae was most closely related to Rubieae among the three representative groups of subfamily Rubioideae.

The genus *Diodia* L. belongs to tribe Spermacoceae, subfamily Rubioideae, family Rubiaceae and consists of about 27 species with accepted names of annual or perennial herbs (The Plant List 2013). The genus is native to warm temperate and tropical America and Africa, with several American species naturalized in the Old World tropics (eFloras 2008). *D. virginiana* L. is the most widely distributed species in the genus (GBIF 2019), has been introduced to and naturalized in Taiwan of China and has not spread beyond the region (eFloras 2008). It is considered a problem weed of lawns and turfgrass by its developed roots and rhizomes throughout the southeastern United States (Graham and Johnson 2004). Now, several plants have been detected at the Jiangsu port, which may have been introduced along with imported grain. Given its invasive features, we should pay enough attention to it in other parts of China besides its naturalization in Taiwan.

On the aspects of phylogenetic relationship, Rubiaceae are currently classified in three subfamilies and more than 40 tribes (Bremer et al. 1995). Tribe Spermacoceae contains at least 1000 species, most of them herbaceous, with pan-tropical distribution and only a few genera in temperate regions. Through the study of Kårehed et al. (2008), it is concluded that Spermacoce is not a monophyletic group, intermingled with several smaller genera of other groups. What is the phylogenetic relationship of Spermacoce in chloroplast genome has not been studied. Therefore, we discussed the phylogenetic positions of Spermacoce and *Diodia* in Rubiaceae and Gentianales on the basis of chloroplast genome represented by *D. virginiana*.

Total DNA (Voucher specimen: 34.746475°N, 119.406474°E, 12198) was isolated using the Plant Genomic DNA Kit (Tiangen Biotech Co., China) and sequenced by the Illumina HiSeq2500 platform (Novogene, Beijing, China). A total of 588,298 paired-end reads were assembled to *Galium aparine* (GenBank accession nr: KY562587.1) and *Dunnia sinensis* (GenBank accession nr: NC039965.1) to produce contigs using Geneious assembler (Biomatters, Auckland, New Zealand).

The total plastome length of *D. virginiana* (MN276038) was 154,387 bp, with large single copy (LSC; 83,625 bp), small single copy (SSC; 17,475 bp), and two inverted repeats (IRa and IRb; 26,643 bp each). The overall GC content was 37.2% (LSC, 34.9%; SSC, 31.2%; IRs, 42.8%) and the plastome contained 137 genes, including 92 protein-coding, eight rRNA, and 37 tRNA genes. A total of 21 genes were duplicated in the inverted repeat regions including seven tRNA, four rRNA, and ten protein coding genes.

To confirm the phylogenetic position of tribe *Spermacoceae*, 24 representative species of Gentianales referring to 3 family, 7 subfamily, 19 tribes were aligned using MAFFT v.7.388 (Katoh and Standley 2013) plug-in Geneious Prime v. 2019.1.3 (Biomatters, Auckland, New Zealand) and Neighber-Joining (NJ) analysis was conducted with *Plagiobothrys nothofulvus* (Boraginales) as an outgroup using Geneious Tree Builder of Geneious Prime v. 2019.1.3 (Biomatters, Auckland, New Zealand) and confidence for nodes determined using bootstrap analysis with 1000 replicates (Figure 1). The NJ consensus tree showed that the 3 families form separate branches, Rubiaceae is monophyletic and has three distinct clades. *Dunnia sinensis* thought to belong to Rubioideae has been considered seriously threatened due primarily to habitat destruction in China, and has not been involved in previous systematic taxonomic studies of the Rubiaceae. Moreover, in line with the previous research, we tend to think that the three subfamilies of Rubiaceae are monophyletic groups respectively. Therefore, whether *Du. sinensis* belongs to Rubioideae or Ixoroideae needs further research. In addition, Cinchonoideae is closely related to Ixoroideae compared with Rubioideae. Spermacoceae was most closely related to Rubieae among the three representative groups of subfamily Rubioideae.

**Figure 1.**
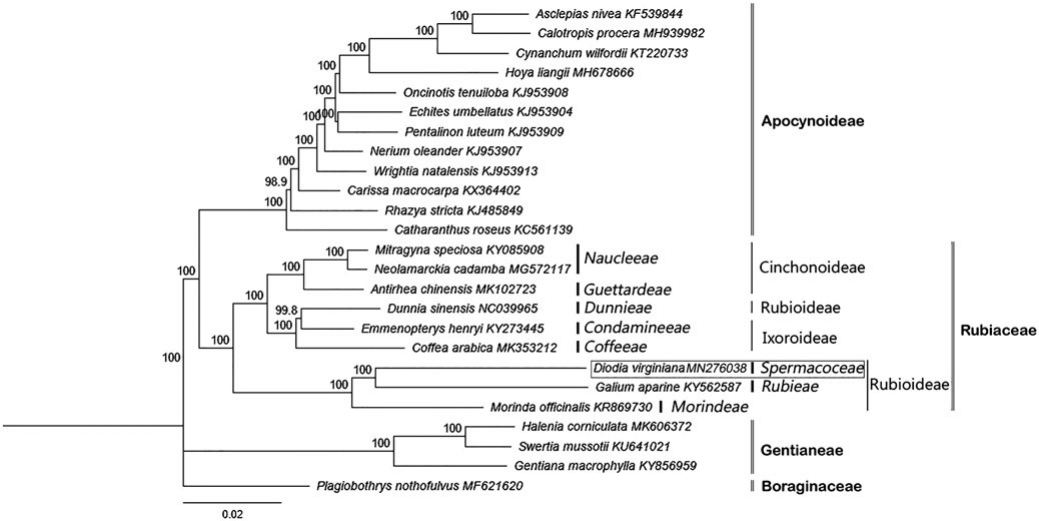
The Neighber-Joining (NJ) tree based on the 24 representative chloroplast genomes of order Gentianales and one outgroup of Boraginales. The bootstrap value based on 1000 replicates is shown on each node.
